# Understanding the Potential Impact of Different Drug Properties on Severe Acute Respiratory Syndrome Coronavirus 2 (SARS-CoV-2) Transmission and Disease Burden: A Modelling Analysis

**DOI:** 10.1093/cid/ciab837

**Published:** 2021-09-21

**Authors:** Charles Whittaker, Oliver J Watson, Carlos Alvarez-Moreno, Nasikarn Angkasekwinai, Adhiratha Boonyasiri, Luis Carlos Triana, Duncan Chanda, Lantharita Charoenpong, Methee Chayakulkeeree, Graham S Cooke, Julio Croda, Zulma M Cucunubá, Bimandra A Djaafara, Cassia F Estofolete, Maria Eugenia Grillet, Nuno R Faria, Silvia Figueiredo Costa, David A Forero-Peña, Diana M Gibb, Anthony C Gordon, Raph L Hamers, Arran Hamlet, Vera Irawany, Anupop Jitmuang, Nukool Keurueangkul, Teresia Njoki Kimani, Margarita Lampo, Anna S Levin, Gustavo Lopardo, Rima Mustafa, Shevanthi Nayagam, Thundon Ngamprasertchai, Ng’ang’a Irene Hannah Njeri, Mauricio L Nogueira, Esteban Ortiz-Prado, Mauricio W Perroud, Andrew N Phillips, Panuwat Promsin, Ambar Qavi, Alison J Rodger, Ester C Sabino, Sorawat Sangkaew, Djayanti Sari, Rujipas Sirijatuphat, Andrei C Sposito, Pratthana Srisangthong, Hayley A Thompson, Zarir Udwadia, Sandra Valderrama-Beltrán, Peter Winskill, Azra C Ghani, Patrick G T Walker, Timothy B Hallett

**Affiliations:** MRC Centre for Global Infectious Disease Analysis, Imperial College London, London, UK; MRC Centre for Global Infectious Disease Analysis, Imperial College London, London, UK; Clínica Universitaria Colombia, Clínica Colsanitas, Facultad de Medicina, Universidad Nacional de Colombia, Bogotá, Colombia; Division of Infectious Diseases and Tropical Medicine, Department of Medicine, Faculty of Medicine Siriraj Hospital, Mahidol University, Bangkok, Thailand; Faculty of Medicine Siriraj Hospital, Mahidol University, Bangkok, Thailand; Hospital Universitario San Ignacio -Pontificia Universidad Javeriana, Bogotá, Colombia; Adult Infectious Diseases Centre, University Teaching Hospital, Lusaka, Zambia; Department of Internal Medicine, University of Zambia School of Medicine, Lusaka, Zambia; Bamrasnaradura Infectious Diseases Institute, Department of Diseases Control, Ministry of Public Health, Nonthaburi, Thailand; Division of Infectious Diseases and Tropical Medicine, Department of Medicine, Faculty of Medicine Siriraj Hospital, Mahidol University, Bangkok, Thailand; Department of Infectious Diseases, Imperial College London, London, UK; NIHR Biomedical Research Centre, Imperial College NHS Trust, London, UK; Oswaldo Cruz Foudantion, Mato Grosso do Sul, Campo Grande, Brazil; School of Medicine, Federal University of Mato Grosso do Sul, Campo Grande, Brazil; Yale School of Public Health, New Haven, Connecticut, USA; MRC Centre for Global Infectious Disease Analysis, Imperial College London, London, UK; Departamento de Epidemiología Clínica y Bioestadística. Facultad de Medicina, Pontificia Universidad Javeriana, Bogotá, Colombia; MRC Centre for Global Infectious Disease Analysis, Imperial College London, London, UK; Eijkman-Oxford Clinical Research Unit, Jakarta, Indonesia; Faculdade de Medicina de São José do Rio Preto (FAMERP), São José do Rio Preto, Brazil; Instituto de Zoologia y Ecologia Tropical, Facultad de Ciencias, Universidad Central de Venezuela, Caracas, Venezuela; MRC Centre for Global Infectious Disease Analysis, Imperial College London, London, UK; Departamento de Molestias Infecciosas e Parasitarias and Instituto de Medicina Tropical da Faculdade de Medicina da Universidade de São Paulo, São Paulo, Brazil; Department of Zoology, University of Oxford, Oxford, UK; Hospital das Clínicas da Faculdade de Medicina da Universidade de São Paulo, São Paulo, Brazil; Biomedical Research and Therapeutic Vaccines Institute, Ciudad Bolívar, Venezuela; MRC Clinical Trials Unit at University College London, London, UK; Division of Anaesthetics, Pain Medicine and Intensive Care, Imperial College London, London, UK; Eijkman-Oxford Clinical Research Unit, Jakarta, Indonesia; Centre for Tropical Medicine and Global Health, Nuffield Dept of Medicine, University of Oxford, Oxford, UK; MRC Centre for Global Infectious Disease Analysis, Imperial College London, London, UK; Fatmawati General Hospital, Faculty of Medicine University of Indonesia, Jakarta, Indonesia; Division of Infectious Diseases and Tropical Medicine, Department of Medicine, Faculty of Medicine Siriraj Hospital, Mahidol University, Bangkok, Thailand; Samutprakan Hospital, Bangkok, Thailand; Kenyan Ministry of Health, Nairobi, Kenya; Instituto Venezolano de Investigaciones Científicas, Caracas, Venezuela; Department of Infectious Diseases, Faculdade de Medicina, Universidade de São Paulo, São Paulo, Brazil; Hospital Bernardo Houssay, Buenos Aires, Argentina; Department of Epidemiology and Biostatistics, Imperial College London, London, UK; MRC Centre for Global Infectious Disease Analysis, Imperial College London, London, UK; Department of Clinical Tropical Medicine, Faculty of Tropical Medicine, Mahidol University, Bangkok, Thailand; Kenyan Ministry of Health, Kiambu County, Kenya; Faculdade de Medicina de São José do Rio Preto (FAMERP), São José do Rio Preto, Brazil; OneHealth Global Research Group, Universidad de las Américas, Quito, Ecuador; School of Medical Sciences; University of Campinas, Campinas, Brazil; Institute for Global Health, University College London, London, UK; Critical Care Division, Department of Medicine, Faculty of Medicine Siriraj Hospital, Mahidol University, Bangkok, Thailand; School of Public Health, Imperial College London, London, UK; Institute for Global Health, University College London, London, UK; Instituto de Medicina Tropical da Faculdade de Medicina da Universidade de São Paulo, São Paulo, Brazil; Section of Adult Infectious Disease, Department of Infectious Disease, Faculty of Medicine, Imperial College London, London, UK; Department of Anesthesiology and Intensive Theraphy, Faculty of Medicine, Public Health and Nursing Universitas Gadjah Mada. Public Hospital Dr. Sardjito, Yogyakarta, Indonesia; Division of Infectious Diseases and Tropical Medicine, Department of Medicine, Faculty of Medicine Siriraj Hospital, Mahidol University, Bangkok, Thailand; Atherosclerosis and Vascular Biology Laboratory, State University of Campinas, Campinas, Brazil; Bangkok Christian Hospital, Bangkok, Thailand; MRC Centre for Global Infectious Disease Analysis, Imperial College London, London, UK; Hinduja Hospital and Research Centre, Mumbai, India; Division of Infectious Diseases. School of Medicine. Pontificia Universidad Javeriana, Hospital Universitario San Ignacio, Bogotá, Colombia; MRC Centre for Global Infectious Disease Analysis, Imperial College London, London, UK; MRC Centre for Global Infectious Disease Analysis, Imperial College London, London, UK; MRC Centre for Global Infectious Disease Analysis, Imperial College London, London, UK; MRC Centre for Global Infectious Disease Analysis, Imperial College London, London, UK

**Keywords:** SARS-CoV-2, COVID-19, epidemiology, therapeutics, modelling

## Abstract

**Background:**

The public health impact of the coronavirus disease 2019 (COVID-19) pandemic has motivated a rapid search for potential therapeutics, with some key successes. However, the potential impact of different treatments, and consequently research and procurement priorities, have not been clear.

**Methods:**

Using a mathematical model of severe acute respiratory syndrome coronavirus 2 (SARS-CoV-2) transmission, COVID-19 disease and clinical care, we explore the public-health impact of different potential therapeutics, under a range of scenarios varying healthcare capacity, epidemic trajectories; and drug efficacy in the absence of supportive care.

**Results:**

The impact of drugs like dexamethasone (delivered to the most critically-ill in hospital and whose therapeutic benefit is expected to depend on the availability of supportive care such as oxygen and mechanical ventilation) is likely to be limited in settings where healthcare capacity is lowest or where uncontrolled epidemics result in hospitals being overwhelmed. As such, it may avert 22% of deaths in high-income countries but only 8% in low-income countries (assuming R = 1.35). Therapeutics for different patient populations (those not in hospital, early in the course of infection) and types of benefit (reducing disease severity or infectiousness, preventing hospitalization) could have much greater benefits, particularly in resource-poor settings facing large epidemics.

**Conclusions:**

Advances in the treatment of COVID-19 to date have been focused on hospitalized-patients and predicated on an assumption of adequate access to supportive care. Therapeutics delivered earlier in the course of infection that reduce the need for healthcare or reduce infectiousness could have significant impact, and research into their efficacy and means of delivery should be a priority.

## Teaser

Coronavirus disease 2019 (COVID-19) drug development to date has focused on reducing deaths among hospitalized patients, but greater public-health impact could come from drugs delivered to outpatients early in the course of disease, and that prevent hospitalization and/or onwards transmission.

The coronavirus disease 2019 (COVID-19) pandemic has led to > 4.5 million deaths as of 1 September 2021 and placed substantial pressure on healthcare systems, with demand for oxygen, advanced respiratory support (ARS), and beds nearing or eclipsing availability in settings hit hardest. This impact has motivated significant efforts to identify and develop therapeutics aimed at treating the disease—a need that has become even greater with the emergence of severe acute respiratory syndrome coronavirus 2 (SARS-CoV-2) variants able to evade prior immunity [1–3]. This has underscored the potential for the virus to become endemic [4] and the need for an integrated, long-term approach to combating COVID-19. Such an approach will require a range of therapeutic options, targeting a range of points across the disease’s natural history.

To date, many clinical trials have been conducted to evaluate potential therapeutics for COVID-19, with initial focus centering on hospitalized patients. Dexamethasone has been shown to reduce mortality in both severely/critically ill [5] and moderately ill patients [6] and is now recommended for use by the World Health Organization (WHO) [7]. Evidence also indicates the potential efficacy of therapeutic anti-coagulation in some patients [8], as well as interleukin-6 receptor antagonists, such as tocilizumab and sarilumab [9]. Other candidates have included antivirals such as remdesivir, although its effect remains uncertain [10, 11]. Recent months have also seen trials focused on individuals who are not hospitalized, including those aiming to prevent progression to hospitalization, such as for colchicine [12] and inhaled-budesonide [13, 14]; as well as molnupiravir [15, 16], peginterferon lambda [17], and monoclonal antibodies [18–20], which may also reduce transmission through reducing viral loads. Numerous other therapeutics aimed at treating early infection in the outpatient setting remain under active development [21].

These therapeutics have diverse epidemiological impacts (reductions in mortality, impacts on healthcare demand, and community transmission) and vary in which patient populations they are administered to (hospitalized individuals or outpatients). Given these diverse properties, understanding the potential impacts of each, and how this is affected by other factors (such as epidemic trajectory and healthcare supply) is vital for guiding procurement and research priorities. Here we use a modeling approach to understand the impact of established and potential COVID-19 therapeutics on disease burden and how this is affected by epidemic context and healthcare resources. Our results highlight how limited healthcare resources can constrain this impact, limiting the benefits of existing therapeutics, and provide insight into the types of therapeutic properties that could be of greatest value.

## METHODS

### Mathematical Model of SARS-CoV-2 Transmission

**Figure 1.**
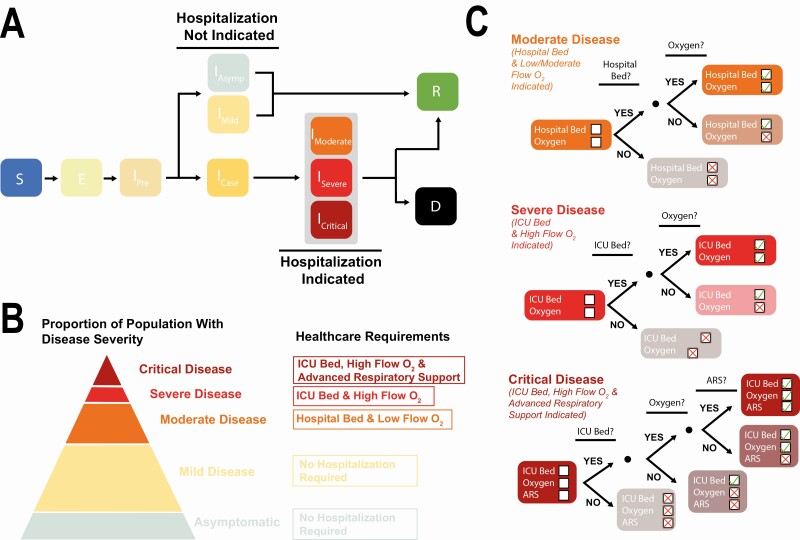
Mathematical modeling approach used to evaluate potential COVID-19 treatment impact. *A*, Schematic representation of the natural history of SARS-CoV-2 infection and COVID-19 disease in the model. *B*, Description of the different disease states included in the model and the associated healthcare requirements. *C*, Decision-tree diagrams illustrating the conditional delivery of healthcare components according to disease severity and availability. There is excess mortality associated with not receiving the full set of required healthcare components. Abbreviations: COVID-19, coronavirus disease 2019; SARS-COV-2, severe acute respiratory syndrome coronavirus 2.

We extended a model of SARS-CoV-2 transmission [22] to include an updated representation of COVID-19 disease, healthcare capacity and the impact of potential therapeutics (Appendix, Figure 1*A*, and Supplementary Figure 1). The model is age-structured and includes a detailed representation of disease severity and clinical care. Those with more serious symptoms deteriorate to the point of requiring hospitalization; they progress to either moderate disease (requiring a general hospital bed and low/moderate-flow oxygen), severe disease (requiring an intensive care unit [ICU] bed and high-flow oxygen) or critical disease (requiring an ICU bed, high-flow oxygen, and ARS) (Figure 1B**).** The model tracks healthcare resource use (beds, oxygen, and ARS devices) to determine what care an individual actually receives (Figure 1C). Individuals recover or die, with a probability determined by an individual’s age, disease severity, and healthcare received (see Appendix for further information).

### Model Parameterization

Natural history parameters for SARS-CoV-2 infection were taken from the literature (Supplementary Tables 1–3). Clinical parameters surrounding duration of hospital stay were derived from a literature review of publications spanning 20 countries (Supplementary Table 4). To derive estimates for parameters not estimable from the literature, we convened a clinical panel of 34 medical professionals who have treated patients with COVID-19 in 11 countries (Argentina, Brazil, Colombia, Ecuador, India, Indonesia, Kenya, Thailand, United Kingdom, Venezuela, and Zambia). This focused on determining the potential effect of dexamethasone under different assumptions of healthcare availability and the overall effect of healthcare resource unavailability (either lack of ARS, oxygen, or beds) on COVID-19 mortality. See Appendix for collated responses.

### Model Simulation

We simulated epidemics under varying degrees of healthcare availability and epidemic trajectories; first in a setting with a profile typical of lower-middle income countries (an age-structure equivalent to the LMIC with the median proportion >65 years and median hospital beds per capita) under 2 epidemic scenarios that reflected different extents of control: a scenario with a high reproduction number for a poorly mitigated epidemic (R = 2), and another with a low reproduction number for a partially mitigated epidemic (R = 1.35). We varied healthcare resource availability, exploring scenarios with (i) unlimited healthcare, (ii) where availability of ARS only is limited, (iii) where ARS and oxygen availability are both limited; and (iv) where ARS, oxygen, and hospital/ICU beds are all limited. To evaluate the potential impact of different therapeutics, we consider 6 different types of therapeutic effects, each corresponding to a mode of action of at least one proposed therapeutic (see Table 1). For country-specific estimation, we fit our model to COVID-19 deaths data [23, 24] using a Bayesian framework (see Appendix) and project the epidemic forwards under different assumptions of future control.

**Table 1.** Potential COVID-19 Therapeutic Effects and Their Impacts

Effect	Description	Target Population	Epidemiological Impact	Examples of Therapeutics Which May Have This Property*	Indicative Potential Efficacy Range	Indicative Potential Coverage Range
Type 1	Reduce COVID-19 mortality	Hospitalized patients (moderately, severely or critically ill)	Reduced mortality	Dexamethasone (moderately [5] and severely/critically ill patients [5, 6]).	20–45% relative reduction in mortality	90–100%
				Remdesivir (moderately ill patients [10, 11], per the meta-analysis of the 2 trials)		
				Tocilizumab and Sarilumab (severely/critically ill patients [9])		
				Therapeutic anticoagulants (moderately ill patients [8])		
Type 2	Reduce COVID-19 severity (in hospitalized patients)	Hospitalized patients (moderately, severely or critically ill)	Reduced mortality and healthcare pressure	Possibly therapeutic anticoagulants (moderately ill patients [8])	20–45% relative reduction in hospitalized patients requiring ICU stay	90–100%
Type 3	Reduce duration of hospitalization with COVID-19	Hospitalized patients (moderately, severely or critically ill)	Reduced healthcare pressure	Remdesivir (moderately ill patients [11]).	20–45% decrease in duration of hospitalization	90–100%
Type 4	Prevent hospitalization due to COVID-19	Post-symptom onset. Mildly symptomatic individuals in the community	Reduced mortality and healthcare pressure	Monoclonal antibodies [18–20]	25–75% reduction in chance of hospitalization	25–50%
				Molnupiravir [15, 16]		
				Inhaled Budesonide [13, 14]		
				Possibly Colchicine [12]		
Type 5a	Reduce duration of infectiousness	Post-symptom onset. Mildly symptomatic individuals in the community	Reduced mortality, healthcare pressure and transmission	Postulated for Monoclonal antibodies due to effect on viral lo-ads [18–20]	25–75% reduction in duration of infectiousness	25–50%
				Possibly Molnupiravir [15, 16]		
				Possibly Peginterferon-Lambda [17]		
Type 5b	Reduce duration of infectiousness	Post-exposure. All individuals exposed to risk of infection, irrespective of symptoms	Reduced mortality, healthcare pressure and transmission	Postulated for Monoclonal antibodies due to effect on viral loads [18–20]	20–75% reduction in duration of infectiousness	10–25%
				Possibly Molnupiravir [15, 16]		
				Possibly Peginterferon-Lambda [17]		

*Inclusion in this list indicates that studies are underway to test for this property and not that evidence has been found.

Abbreviation: COVID-19, coronavirus disease 2019.

## RESULTS

**Evaluating the Impact of Dexamethasone Under Different Assumptions of Epidemic Spread and Health System Capacity**

**Figure 2.**
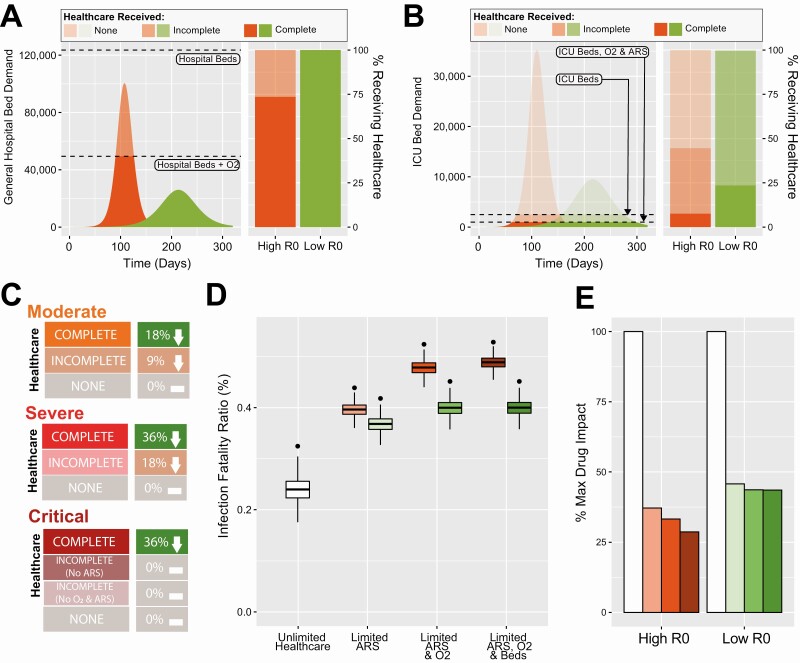
Projected impact of dexamethasone on COVID-19 mortality under different scenarios of epidemic progression and healthcare availability. *A*, Daily general hospital bed demand under an epidemic scenario with a high reproduction number (R = 2, orange) or a low reproduction number (R = 1.35, green). Dashed lines indicate availability of different healthcare resources, and the right hand panel describes the proportion of patients that require oxygen and a general hospital bed who receive complete (bed and oxygen), incomplete (bed only) or no healthcare (neither). *B*, As in panel (*A*) but describing demand and healthcare received for severely and critically ill patients requiring an ICU bed, oxygen, and ARS. *C*, Schematic illustration of the impact assumed for dexamethasone on COVID-19 mortality in different patient populations (moderate, severe or critical illness), and according to the care received (complete, incomplete or none). *D*, Impact of dexamethasone on the COVID-19 infection fatality ratio under different assumptions for R (low, green or high, orange) and healthcare availability (unlimited, limited ARS, limited ARS and oxygen or limited ARS, oxygen and beds). In all panels, black points show the IFR without dexamethasone, and the boxplots show the modelled IFR using the assumed dexamethasone clinical benefit estimates described in panel (*C*). *E*, Percentage of maximum potential dexamethasone impact (defined as the reduction in IFR achieved by dexamethasone under a situation of unlimited healthcare) achieved in each of the different scenarios for healthcare availability. Orange and green bars refer to high and low R scenarios, respectively, with the shading indicating the extent of imposed healthcare constraints, colored as for panel (*D*). Abbreviations: ARS, advanced respiratory support; COVID-19, coronavirus disease 2019; ICU, intensive care unit; IFR, infection fatality ratio.

We simulated an epidemic in a setting with a profile typical of LMICs under two epidemic scenarios (R = 1.35 or 2.0). Our results highlight the substantial difference in the timing and intensity of healthcare demand resulting from epidemics of different sizes. Higher R epidemics (R = 2, representing a poorly mitigated epidemic) lead to a smaller fraction of moderately ill patients (requiring a general hospital bed, Figure 2A) and severely/critically ill patients (requiring ICU-based care, Figure 2B) receiving the clinical care they need, with this disparity most pronounced for ICU-based care. A lower R reduces demand for healthcare, resulting in a higher proportion of individuals receiving the required care but still leaves a high proportion not receiving the full ICU-based care they need. We next examine the “infection fatality ratio” (IFR, the probability of death given infection) that persons with SARS-CoV-2 face, rising from the joint effect of disease, healthcare capabilities, and usage of dexamethasone. Our results highlight the pronounced impact of healthcare constraints on the IFR, which is significantly higher when healthcare resources (ARS, O_2_, and beds) are limited (Figure 2D). This increase in IFR is most substantial for our high R scenario in which a higher fraction of individuals not receiving adequate care.

The therapeutic impact of dexamethasone (Figure 2D, *boxes*) is strongly dependent on these same factors: there is a substantial reduction in mortality due to the drug when there are adequate healthcare resources, but a much smaller effect when these resources are unavailable. This is especially the case when a larger epidemic has overwhelmed resources (Figure 2D). The reduced impact of dexamethasone in these circumstances is because fewer individuals are hospitalized and receive dexamethasone (due to shortages of beds) and fewer hospitalized individuals receive the other healthcare required (oxygen/ARS) to maximize the therapeutic benefit of dexamethasone. As a result, prevailing healthcare resources in this typical setting allow only 45% (if R = 1.35) or 28% (if R = 2.0) of the maximum potential impact of dexamethasone (defined as the reduction in IFR achieved by the drug under a scenario with no healthcare resource constraints) to be realized (Figure 2E).

We distinguish 2 layers of uncertainty in characterizing the effect of dexamethasone: the magnitude of the effect when supportive care is available, and the extent to which these effects would persist in patients not receiving such care. The second is not well understood but we constructed three alternative scenarios (based on clinical input described in the Supplementary Materials) for the extent to which patients without supportive care may benefit from dexamethasone. We find only a small extra impact (60% of potential drug impact realized under R = 1.35 scenario, and 52% under R = 2 scenario) if it was assumed that individuals for whom supportive care could not be provided still benefited to some degree from dexamethasone (Supplementary Figure 3).

**Evaluating the Potential Impact of Dexamethasone Globally**

**Figure 3.**
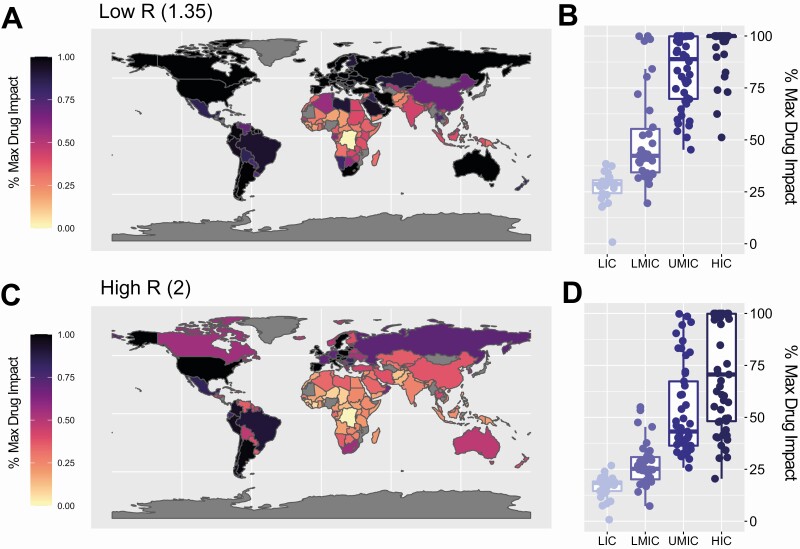
Global impact of dexamethasone on COVID-19 mortality under different assumptions for future transmission and epidemic spread. *A*, Percentage of maximum potential dexamethasone impact (defined as the reduction in IFR achieved by dexamethasone under a situation of unlimited healthcare) achieved for each country under an epidemic scenario of extensive mitigation control (R = 1.35). *B,* Percentage of maximum dexamethasone impact achieved in each country. Each dot is the result for a single country, colored according to the World Bank strata that country belongs to, with the boxplot presenting summary statistics for the modelled countries in aggregate. (C) As in panel (*A*), under an assumption of an epidemic scenario characterized by uncontrolled spread (R = 2). (D) As in panel (*B*), under an assumption of an epidemic scenario characterized by uncontrolled spread (R = 2). Abbreviations: COVID-19, coronavirus disease 2019; IFR, infection fatality ratio.

Our results suggest that limitations in healthcare capabilities that reduce dexamethasone’s impact are likely to be most severe in LMICs. Under scenarios where extensive mitigation of transmission is achieved globally (R = 1.35 scenario, Figure 3A), we expect a median of 28%, 43%, 91%, and 100% of dexamethasone’s maximum potential impact to be achieved across LICs, LMICs, UMICs, and HICs, respectively (Figure 3B), corresponding to averting 8%, 13%, 20%, and 22% of total deaths. Under scenarios where epidemics are less controlled (R = 2, Figure 3C), this reduces to 18%, 26%, 43%, and 71% of dexamethasone’s maximum potential impact (5%, 7%, 13%, and 18% of deaths averted) (Figure 3D).

**Exploring the Potential Impact of Different Treatments and Drug Properties**

**Figure 4.**
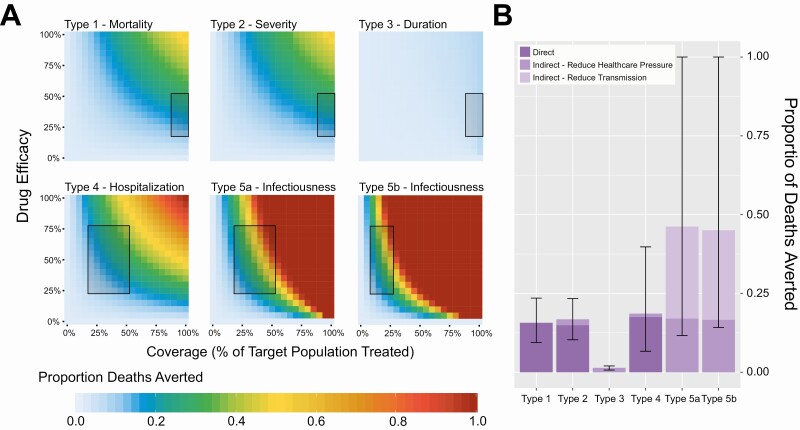
Impact of different therapeutic product effects on COVID-19 disease burden. *A*, For an epidemic with an R of 1.35, the proportion of COVID-19 deaths averted as a function of therapeutic efficacy and therapeutic coverage, for 6 different types of potential effects (Table 1). These include reducing COVID-19 disease mortality (Type 1); preventing deterioration and worsening of disease in hospitalized patients (Type 2); reducing duration of hospitalization (Type 3); preventing hospitalization due to COVID-19 (Type 4) and reducing duration of infectiousness, either among symptomatic (Types 5a) or all infected-persons (Type 5b). Inset boxes indicate the range of plausible values of coverage used to generate the estimates in panel (*B*). *B*, Disaggregation of therapeutic effect type impact by whether this is direct or indirect. Bars are colored according to the type of impact (direct reduction in mortality, indirect reduction in mortality due to reduced pressure on healthcare or indirect reduction in mortality due to reductions in community transmission), with error bars indicating the maximum and minimum proportion of deaths averted under the range of coverage and effectiveness values considered for each effect type (indicated by the boxes in panel (*A*) and Table 1). Abbreviations: COVID-19, coronavirus disease 2019; IFR, infection fatality ratio.

We divide the spectrum of potential effects of the therapeutics currently under investigation into six types (Table 1) and explore their impact on COVID-19 mortality (Figure 4A and Supplementary Figure 4). The impact of therapeutics administered to hospitalized patients (types 1, 2, and 3) have a lower overall impact in reducing deaths, even when efficacy and coverage are high, because they suffer from the limitation that their therapeutic benefit is dependent on similar healthcare capabilities (such as beds, oxygen and ARS) described above for dexamethasone (Figure 4A, *top row*). Therapeutics that reduce severity of disease (Type 2) or reduce the duration of hospitalization (Type 3) do have an indirect effect in alleviating healthcare demand, but this is minimal because demand for healthcare resources outstrips supply by such a wide margin, even under comparatively well-controlled epidemics (low R scenario). In these scenarios, a slightly faster throughput of patients therefore does not substantially reduce the number of individuals unable to access healthcare due to a lack of availability.

Therapeutics that are not administered in hospitals (and so do not suffer the same limitations) and address patients at an earlier stage of disease progression have a potentially greater impact, even after allowing for the lower coverages that may be achieved (Figure 4A, *bottom row*). Type 4 therapeutics (which reduce likelihood of severe disease and hospitalization) have both a direct effect (reducing mortality) and an indirect effect (reducing healthcare demand and enabling greater access to healthcare for others) avert a significant fraction of COVID-19 mortality. For our low R scenario, there is an even greater effect from Type 5 therapeutics (which reduce infectiousness). Through reducing community transmission, they lead to reductions in the overall number of people infected with SARS-CoV-2 during the epidemic and alleviate demand for healthcare resources. This would be especially the case if therapeutics are administered before onset of symptoms (Type 5b), although the coverage that could be achieved with such therapeutics would be expected to be lower than for therapeutics administered following symptom onset (Type 5a). It follows that the estimates of impact are influenced by R and healthcare resources (see Supplementary Figure 4 in SI): for Type 5 therapeutics, relative impact is higher under the low R scenario and lower in the high R scenario (although still comparable with the best performing hospital administered therapeutics); for our high R scenario, Type 4 therapeutics were predicted to have the greatest benefit in the range of indicative coverages and efficacies explored (SupplementaryFigure 4B**).** If healthcare needs do not eclipse resources, the direct effect of hospital-delivered therapeutics is greater than otherwise, although the overall impact on mortality from Types 4, 5a, and 5b remains high.

## DISCUSSION

Understanding the contexts in which COVID-19 treatments are likely to be most effective is essential for guiding research and procurement. Here we utilize a modeling approach to evaluate the potential impact of COVID-19 treatments under a range of different assumptions about healthcare availability and epidemic trajectory. Our results show that effect sizes for therapeutics estimated in clinical trials will not necessarily provide a guide to their “real-world” impact on COVID-19 disease burden as “real-world” impact also crucially depends on prevailing healthcare constraints, the trajectory of the epidemic and the extent to which benefits persist in the absence of supportive care. We find that the impact of the main therapeutic currently recommended by the WHO (dexamethasone) could be considerable in well-resourced settings with an epidemic under control (averting almost a quarter of deaths) but far smaller in settings where resources are limited and/or there is large epidemic (averting fewer than 10% of deaths). Although our focus here is on dexamethasone, these results would apply similarly to other therapeutics for which clinical benefit is dependent on the presence of supportive care such as oxygen or ARS.

Our results highlight that treatments with different types of effect can yield vastly different scales of population-level impact. In particular, the results show that substantial impact could be achieved with therapeutics delivered to persons not in hospital that either reduce the duration of infectiousness (and hence transmission) or disease severity (preventing hospitalization, reducing healthcare strain), in keeping with recent work highlighting the need for effective COVID-19 treatment for early infection in the outpatient setting [21]. Indeed, our results highlight that even modest levels of treatment efficacy or coverage could achieve high levels of impact, although the exact level of impact will likely be determined by a complex interplay of baseline transmission, household structure, quarantining practices, and the background of other control measures being implemented—factors only crudely considered here through our modulation of the reproduction number. However, because of the nature of their administration (delivered in the community) and the effects of these therapeutics (which depend only minimally on the availability of constrained healthcare resources), our results suggest their potential impact would also be less affected during larger epidemics.

Although most trials to date have focused on evaluating treatments aimed at critically ill, hospitalized patients, there are promising results from some trials. Several individual/combination monoclonal antibody treatments have shown an impact on viral loads and hospitalization [18–20]; however, significant challenges related to delivery (the need for intravenous infusions) and their high cost likely preclude widespread utilization in resource-poor settings. Numerous repurposed therapeutics have also been or are currently being evaluated as part of large-scale adaptive trials: these include PRINCIPLE (evaluating azithromycin [25], doxycycline [22], and inhaled budesonide [13] in outpatient populations, among other drugs), ANTICOV (led by the Drugs for Neglected Diseases Initiative, evaluating a number of different therapeutics in 13 countries across Africa [26]), and the ACTIV-6 platform, which is testing a number of repurposed drugs [27]. Although some of these (eg, inhaled budesonide) have shown promise, the majority of drugs assessed through these platforms aim to reduce duration and severity of symptoms in those with mild disease (a Type 4 property), rather than transmission (Type 5). It is in this context that results from the trials of orally administered antiviral molnupiravir (which has shown preliminary evidence of potentially both properties) are eagerly anticipated [15, 16].

Although the impact of drugs delivered in the outpatient setting are less dependent on prevailing in-hospital healthcare resources, this would need to be balanced by the ability of healthcare systems to deliver therapeutics in the community (including health worker capacity and distribution channels) and the costs of doing so. There thus remain numerous factors that will modulate their effectiveness that warrant discussion here. Perhaps most crucially is the need for rapid, widely available COVID-19 testing to identify persons infected early and hence maximize reductions in onward transmission achieved by drugs with Type 5 properties (and to a lesser degree, ensuring Type 4 drugs are delivered to individuals before significant disease progression). Testing capacity thus represents a crucial determinant of the effectiveness of these drugs, but this remains inadequate in many parts of the world: for instance, recent results from a post-mortem surveillance study in Lusaka, Zambia, suggest that the majority of COVID-19 deaths (>70%) had occurred without any test having been conducted Mwananyanda et al. (https://www.bmj.com/content/372/bmj.n334). A related limitation is that we assume levels of healthcare-seeking within the population such that all individuals with COVID-19 requiring hospitalization will seek care. Numerous studies have highlighted the disparities in access to healthcare that exist globally (eg, [28, 29]), and that cost of care (if borne privately) can be a key determinant [30]. To the extent that not all of those in need seek care in-hospital, the limitations we have found for therapeutics for hospitalized patients and the potential benefits of therapeutics for nonhospitalized patients would be even greater than our results show. More generally, whilst our results have highlighted that only modest levels of coverage among patient populations with these therapeutics is required for significant impact, such levels are likely impractical for therapeutics requiring infusion such as monoclonal antibody therapies. The current cost of these therapies is also substantial and may prove prohibitive in all but the most well-resourced settings. Achieving levels of coverage required for substantial impact may be more feasible for orally delivered, low-cost therapeutics.

Additional caveats to the framework developed here includes lack of waning immunity or the possibility of novel SARS-CoV-2 variants able to partially evade protective immunity (as in Brazil [2], South Africa [31], and many other countries), nor how emergence of new variants may erode efficacy of previously effective therapeutics (such as bamlanivimab in the case of the Delta [32]). We further consider only country-level outcomes, which indicates broad trends but which masks important sub-national variation in the availability of healthcare resources (eg, as highlighted in recent work across Indonesia [33] and Brazil [34]) that would likely see mortality concentrated in areas with the least capacity—nor do we take into account the COVID-19 death underreporting that is likely concentrated in resource-poor settings with the least developed civil and vital registration capacity, and would result in inference of more mature epidemics and higher degrees of population-level immunity. The modeling also does not consider the judgments that may be made about how available resources are allocated among different patients, in view of their varying needs, risk of complications (including “long COVID”), and likelihood of success of different treatment options, which may mitigate to some small extent that effect of the constraints indicated.

Despite these caveats, our results highlight that low health system capacity in LMICs will likely limit the impact of many of the COVID-19 therapeutics currently being used to treat hospitalized patients (such as dexamethasone) and underscore the crucial need for effective COVID-19 therapeutics targeting outpatients with mild-to-moderate disease, early in the disease course. However, we also highlight important logistical and practical challenges to achieving the significant impact possible with these therapeutics, underscoring the importance of accompanying clinical trials with operational research in order to ensure mechanisms for drug delivery to affected communities can occur in a way that maximizes their potential benefit.

## Supplementary Data

Supplementary materials are available at *Clinical Infectious Diseases* online. Consisting of data provided by the authors to benefit the reader, the posted materials are not copyedited and are the sole responsibility of the authors, so questions or comments should be addressed to the corresponding author.

ciab837_suppl_Supplementary_Materials

## Notes

***Contributions.*** C. W. and T. B. H. conceived of the study. C. W., O. J. W., and P. G. T. W. undertook the modeling and data analysis, with input from T. B. H., A. C. G., A. H., H. T., P. W., and A. Q. All other authors contributed to forming modeling assumptions and interpreting results in a clinical panel or assisted in the setting up of the clinical panel. C. W. and T. B. H. produced the first draft of the manuscript. All authors contributed to the final draft.

***Role of the funding source.*** The funder of the study had no role in study design, data collection, data analysis, data interpretation, or writing of the report.

***Financial sources.*** C. W. acknowledges funding from a Medical Research Council (MRC) Doctoral Training Partnership PhD Studentship, as well as funding from the MRC Centre for Global Infectious Disease Analysis (reference MR/R015600/1), jointly funded by the UK Medical Research Council (MRC) and the UK Foreign, Commonwealth and Development Office (FCDO), under the MRC/FCDO Concordat agreement and is also part of the EDCTP2 program supported by the European Union. P. G. T. W., O. J. W., and A. C. G. acknowledge grant funding from The Wellcome Trust and the UK Foreign, Commonwealth and Development Office (FCDO) (reference 221350/Z/20/Z). N. R. F. acknowledges Medical Research Council-São Paulo Research Foundation (FAPESP) CADDE partnership award (MR/S0195/1 and FAPESP 18/14389-0) (https://caddecentre.org). Wellcome Trust and Royal Society (Sir Henry Dale Fellowship: 204311/Z/16/Z). P. W. reports receiving grant (paid to institution) Modelling the Impact of COVID-19 Vaccination Strategies from World Health Organization (WHO); grant (paid to institution) Regional Support to Address the Outbreak of Coronavirus Disease 2019 and Potential Outbreak of Other Communicable Diseases from Asian Development Bank (ADB); Consultancy (3 days) for COVID-19 vaccine modelling support for WHO EURO, outside the submitted work. P. W. reports grant funding from The Wellcome Trust and the UK Foreign, Commonwealth & Development Office (FCDO) (reference 221350/Z/20/Z), during the conduct of the study. G. C. reports NIHR Research Professorship (2018–date); Wellcome Trust Collaborative Award (2017–date); Site for GS5774/5773 trials of remdesivir (2020); serving as Chair WHO Committee on Selection and Use of Essential Medicines, outside the conduct of the study. J. C. reports support from the National Institutes of Health (NIH), biomerieux, Gates Foundation, and Sanofi-Aventis Farmacêutica, and consulting fees from Merck, outside the submitted work. A. C. Gh. reports support from MRC, Wellcome Trust, and UK Foreign, Commonwealth & Development Office (FCDO) to their institution, during the conduct of the study; support from Bill & Melinda Gates Foundation (BMGF) and NIH to their institution; consulting fees from HSBC (Personal [1 hour advice on global pandemic]) and The Global Fund (Personal [non-COVID work]); personal honoraria from US Government; serving in leadership/fiduciary roles for the following: Moderna (Advisory board—non-renumerated), CEPI (Advisory board—non-renumerated), Wellcome Trust (Science interview panel—personal), and Malaria No More UK (Trustee, nonrenumerated), outside the conduct of the study. A. C. Go. reports NIHR Research Professorship (RP-2015-06-18), during the conduct of the study; support from NIHR (REMAP-CAP trial grant) and European Union FP7-HEALTH-2013-INNOVATION-1 (grant number 602525); participation on Advisory Board/DSMB for 30 Respiratory (paid to institution). Z. F. U. has been Principal Investigator in a trial on Favipravir supported by Glenmark Pharmaceuticals (no funds/payments received), during the conduct of the study. T. H. reports that the study was supported by the MRC Centre for Global Infectious Disease Analysis and that no other funding was provided for this work specifically.

***Potential conflicts of interest.*** The authors: No reported conflicts of interest. All authors have submitted the ICMJE Form for Disclosure of Potential Conflicts of Interest. Conflicts that the editors consider relevant to the content of the manuscript have been disclosed. None of the authors have potential conflicts.
